# Lesions in the right Rolandic operculum are associated with self-rating affective and apathetic depressive symptoms for post-stroke patients

**DOI:** 10.1038/s41598-020-77136-5

**Published:** 2020-11-20

**Authors:** Stephanie Sutoko, Hirokazu Atsumori, Akiko Obata, Tsukasa Funane, Akihiko Kandori, Koji Shimonaga, Seiji Hama, Shigeto Yamawaki, Toshio Tsuji

**Affiliations:** 1 Center for Exploratory Research, Research and Development Group, Hitachi. Ltd., Tokyo, Japan; 2 Department of Rehabilitation, Hibino Hospital, Hiroshima, Japan; 3 Department of Neurosurgery and Interventional Neuroradiology, Hiroshima City Asa Citizens Hospital, Hiroshima, Japan; 4 Department of Neurosurgery, Graduate School of Biomedical and Health Sciences, Hiroshima University, Hiroshima, Japan; 5 Center for Brain, Mind and KANSEI Sciences Research, Hiroshima University, Hiroshima, Japan; 6 Graduate School of Advanced Science and Engineering, Hiroshima University, Hiroshima, Japan

**Keywords:** Neuroscience, Psychology, Biomarkers, Diseases, Medical research, Neurology

## Abstract

Stroke survivors majorly suffered from post-stroke depression (PSD). The PSD diagnosis is commonly performed based on the clinical cut-off for psychometric inventories. However, we hypothesized that PSD involves spectrum symptoms (e.g., apathy, depression, anxiety, and stress domains) and severity levels. Therefore, instead of using the clinical cut-off, we suggested a data-driven analysis to interpret patient spectrum conditions. The patients’ psychological conditions were categorized in an unsupervised manner using the *k*-means clustering method, and the relationships between psychological conditions and quantitative lesion degrees were evaluated. This study involved one hundred sixty-five patient data; all patients were able to understand and perform self-rating psychological conditions (i.e., no aphasia). Four severity levels—low, low-to-moderate, moderate-to-high, and high—were observed for each combination of two psychological domains. Patients with worse conditions showed the significantly greater lesion degree at the right Rolandic operculum (part of Brodmann area 43). The dissimilarities between stress and other domains were also suggested. Patients with high stress were specifically associated with lesions in the left thalamus. Impaired emotion processing and stress-affected functions have been frequently related to those lesion regions. Those lesions were also robust and localized, suggesting the possibility of an objective for predicting psychological conditions from brain lesions.

## Introduction

Structural brain abnormalities caused by brain infarction and hemorrhage bring complex impairments related to physical-cognitive functions and psychological conditions. Post-stroke depression (PSD) is closely linked to (affective) depression and apathy symptoms. Patients lacking self-acceptance due to stroke-evoked disabilities and having irrational expectations of their recovery course (high insistence on recovery) may develop both symptoms. Even though both result in negative effects on a patient’s quality of life^1,2^, those symptoms are considered distinct and separate domains. A previous study based on computed tomography imaging reported that lesion location may affect different symptoms. The severity of depression was related to lesions in the left frontal lobe; the symptomatic apathy was associated with the damage of bilateral basal ganglia^3^. Those symptoms affected serotonergic and dopaminergic neurotransmitter pathways differently^4^, and effects of those symptoms on lesions were also less likely overlapped (12–21%; brainstem lesions). The improvement of physical abilities was claimed to reduce depressive and apathetic symptoms^2^. However, the apathy domain was a better predictor of functional recovery than the depressive symptoms^5^.

More than 50% of reported stroke patients suffered from apathy and/or depression, with varied possibilities of apathy without depression (20–28%), depression without apathy (12–20%), and both psychological symptoms (15–21%) across sites^2–4^. The variations of psychological condition and even affected left hemispheric lesion might be caused by methodological differences across studies^6^. The diagnostic discrepancy is likely to have been a result of using divergent types of psychometric inventories with particular cut-offs. Furthermore, the applied cut-off interpreted psychological condition from a binary perspective of severity level (diagnosed vs. non-diagnosed; low vs. high). Here, however, we argue for a higher-order interpretation on the basis of multiple severity levels (i.e., a spectrum) of apathetic-depressive symptoms or even other psychological domains (e.g., anxiety, stress).

As discussed above, the implementation of cut-offs on patient’s scores of psychometric inventories may bring about a limited interpretation for the dataset. Therefore, rather than using the pre-set clinical cut-offs, we suggest data-driven and unsupervised categorization of patient data based on two psychological domains. The existent of lesions had been associated with psychological conditions as mentioned above, but the relationship between a spectrum of severity levels and quantitative lesion degrees has not been studied before. Therefore, in the current study, we aimed to understand the spectrum of psychological domains and severity levels for stroke patients and its relationships with brain lesion degrees.

## Results

### Four clusters interpreting psychological conditions for stroke patients

**Figure 1:**
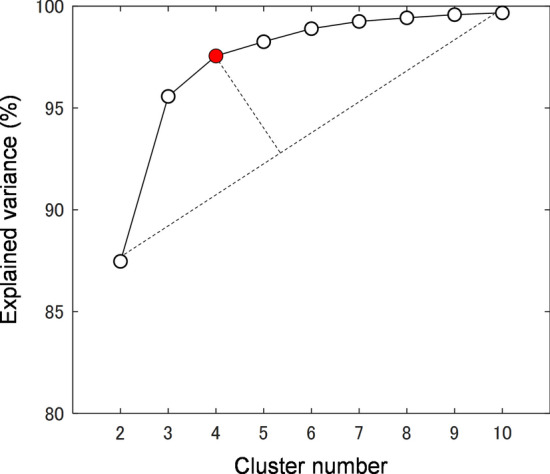
Plot of explained variance against cluster number in categorizing patient data based on their apathy and depression scores. By using the elbow method, the optimum cluster number was found to be four.

Unsupervised categorization requires the pre-determined cluster number. In order to avoid any assumptions, the cluster number was optimized. The optimization target was the clustering efficiency evaluated by a parameter, namely, explained variance (range 0–100%). As the cluster number increases, the explained variance parameter gradually increases and reaches a plateau as shown in Fig. 1. The difference of explained variance for two sequential cluster numbers was initially high (for the increase from two clusters to three) and gradually decreased (for the increases from three clusters to four, four to five, and so on). The smallest cluster number (i.e., two clusters) revealed around 87% explained variance; the largest cluster number (i.e., ten clusters) brought almost 100% explained variance. When the increase of explained variance becomes insignificant, further increasing the cluster number will not result in better clustering. In order to determine the optimum cluster number, the elbow method^7,8^ was applied on the plot of explained variance against cluster number. A first-degree line between the most-distant points (two and ten clusters) was fitted. The distance between points ([cluster number, explained variance]; red dots in Fig. 1) and the line was computed. The longest distance was obtained by the point of four clusters (98% explained variance). Therefore, four was selected as the optimum cluster number. The optimum cluster number for classifying patient data based on other combinations of psychological domains (e.g., apathy–anxiety, depression–anxiety, and so on) was also four clusters (data not shown).

### Distinct lesion characteristics for each cluster

**Figure 2:**
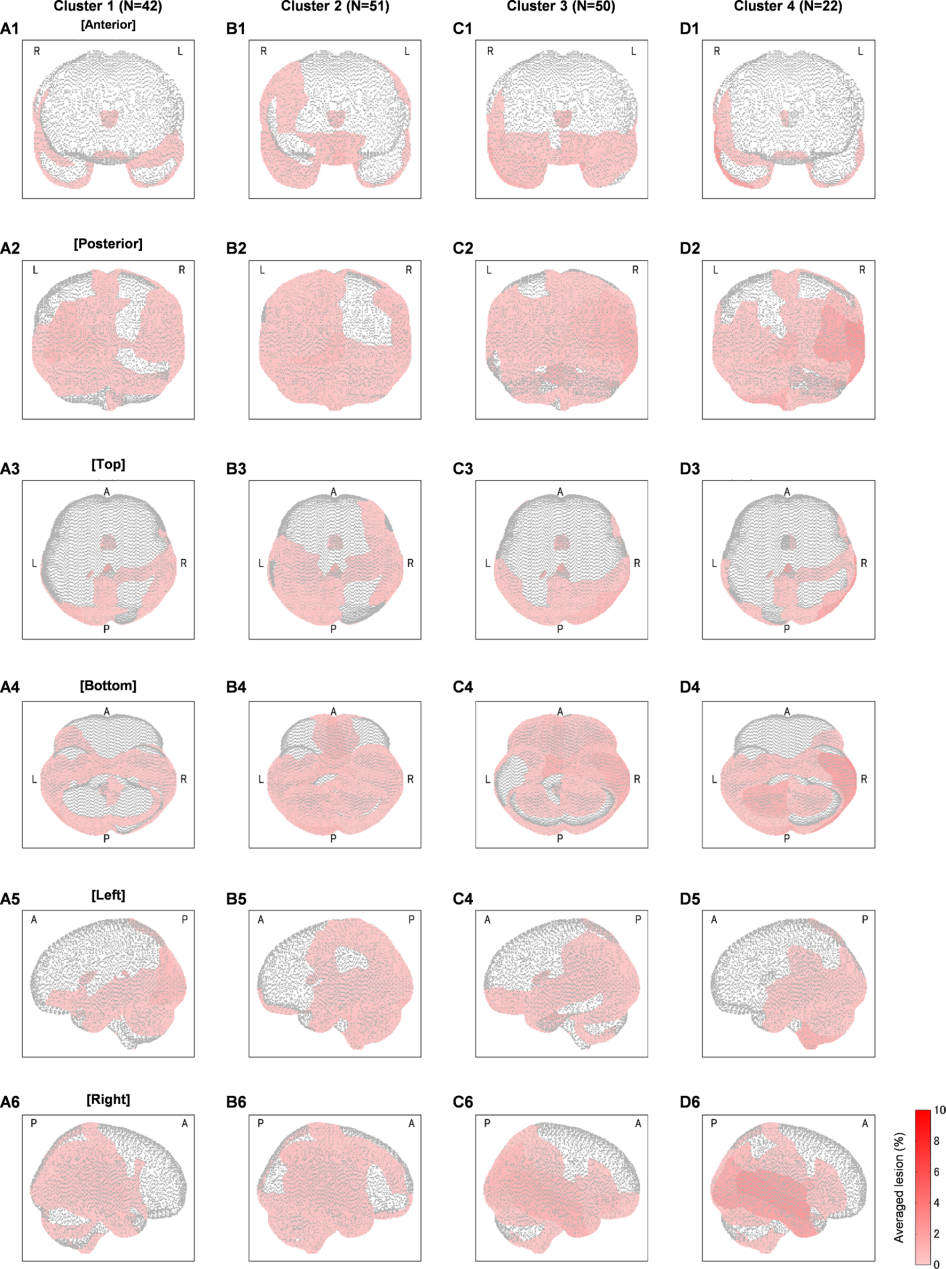
Lesion maps for each clusters (**A**–**D** for clusters 1, 2, 3, and 4, respectively) visualized in anterior (**A1**–**D1**), posterior (**A2**–**D2**), top (**A3**–**D3**), bottom (**A4**–**D4**), left (**A5**–**D5**), and right (**A6**–**D6**) views. Color bar represents the lesion degree in percentage. A, P, R, and L denote anterior, posterior, right, and left, respectively.

The patient data on depression-plotted-against-apathy scores were categorized into four clusters. Forty-two, 51, 50, and 22 patient data were categorized for clusters 1, 2, 3, and 4, respectively. Figure 2 shows the lesion maps visualized in six ways—anterior, posterior, top, bottom, left, and right views. The colored patches on brain identify the lesion degrees averaged across patients. These maps improved the process of exploratory data analysis. The lesion characteristics were distinct for each cluster. While all clusters revealed major lesions in the occipital lobe (Fig. 2A2–D2), the prefrontal lesions were observed only for clusters 2 and 3 (Fig. 2B1, C1). Cluster 2 was distinguished from cluster 3 based on lesions found in the left precentral–postcentral–superior parietal gyri (Fig. 2B3 vs. C3). Clusters 1 and 4 revealed lesions in the orbital part of inferior frontal gyrus with specific left (Fig. 2A4) and right (Fig. 2D4) laterality for respective clusters. Besides the lesion locations, the lesion degree (percentage) may be useful to explain cluster characteristics. For example, even though both clusters 1 and 4 showed lesions in the middle and inferior temporal gyri, a greater lesion degree was observed for cluster 4 (Fig. 2C6 vs. D6).

### Cluster effect on brain lesion degree at the right Rolandic operculum and the left thalamus

**Figure 3:**
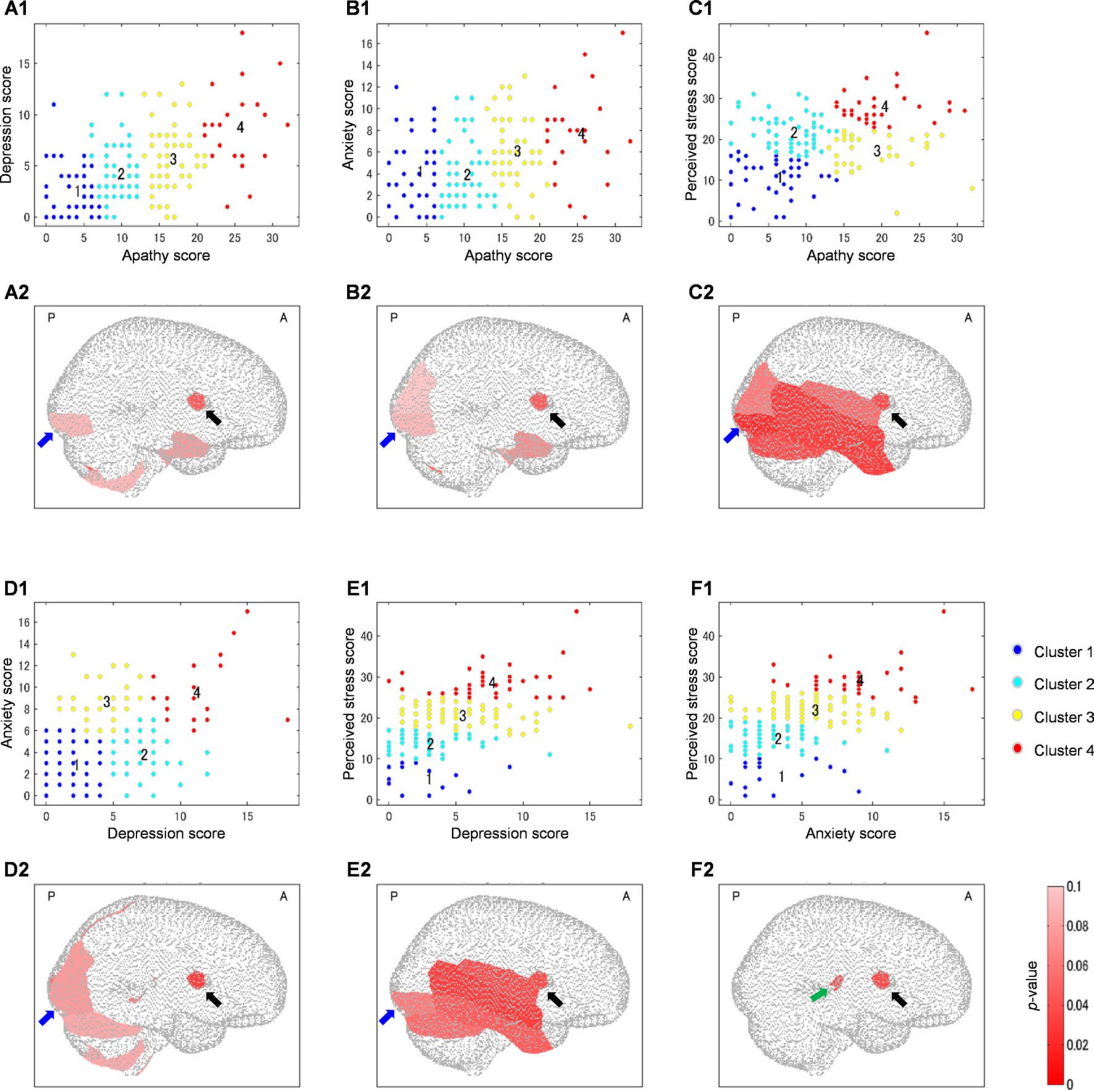
Psychological characteristics (**A1**–**F1**) for each cluster based on apathy–depression (**A**), apathy–anxiety (**B**), apathy–perceived stress (**C**), depression–anxiety (**D**), depression–perceived stress (**E**), and anxiety–perceived stress (**F**) clustering. Differences between clusters are also displayed in *p* value maps (**A2**–**F2**). Black, blue, and green arrows indicate lesions in the right Rolandic operculum, right inferior occipital gyrus, and left thalamus.

The psychological domains are displayed in 2-axis scatter plots in which each axis represents a psychological domain (Fig. 3). There are two clustering characteristics. *First*, each psychological domain was categorized into four severity levels, such as low, low-to-moderate, moderate-to-high, and high. Clusters then characterized the same level for both psychological domains (low apathy and low depression, high apathy and high depression; clusters 1 and 4 in Fig. 3A1). This clustering characteristic was found in the combined domains of apathy–depression (Fig. 3A1), apathy–anxiety (Fig. 3B1), depression–perceived stress (Fig. 3E1), and anxiety–perceived stress (Fig. 3F1). *Second*, each psychological domain was categorized into three severity levels, such as low, moderate, and high. While two clusters represented the same level for both psychological domains (e.g., low apathy and low perceived stress, high apathy and high perceived stress; Fig. 3C1), two other clusters were specified by the moderate-low (moderate apathy and low perceived stress; cluster 3; Fig. 3C1) and low-moderate (low apathy and moderate perceived stress; cluster 2; Fig. 3C1) levels for psychological domains. A similar clustering characteristic was observed in the combined parameters of apathy–perceived stress (Fig. 3C1) and depression–anxiety (Fig. 3D1).

The cluster number had been ordered depending on the levels of psychological domains. The most-distant clusters (i.e., clusters with low and high psychological scores; clusters 1 and 4) were noticeable; the in-between clusters (i.e., clusters with low-to-moderate and moderate-to-high psychological scores) were determined by the distance between those centroids and the centroids of most-distant clusters. The cluster having a centroid closer to the centroid of the cluster with high psychological scores was assigned to the moderate-to-high cluster (i.e., cluster 3), and vice versa for the low-to-moderate cluster (i.e., cluster 2). Furthermore, the comparisons between clusters were assessed using a one-way analysis of variance (ANOVA). The significant results were visualized in *p* value maps on the brain template (Fig. 3A2–F2). There are three points highlighted from these results. *First*, a significant difference (one-way ANOVA, *F*_*(3,161)*_ = 2.95–4.26, *p* < 0.05) between clusters was observed for lesions in the right Rolandic operculum (black arrows; Fig. 3A2–F2). Lesions in the right inferior occipital gyrus (blue arrows; Fig. 3A2–E2) also frequently brought significant differences between clusters (one-way ANOVA, *F*_*(3,161)*_ = 2.19–4.68, *p* < 0.1). *Second*, the cluster effects on lesions in the lobule VIIB of left cerebellum (one-way ANOVA, *F*_*(3,161)*_ = 2.35–2.99, *p* < 0.1) were distinctly associated with the depression and anxiety scores. *Third*, the lesions differing significantly between clusters specified by the perceived stress score (one-way ANOVA, *F*_*(3,161)*_ = 2.40–3.25, *p* < 0.1) were found in the left thalamus (green arrows; Fig. 3F2).

**Figure 4:**
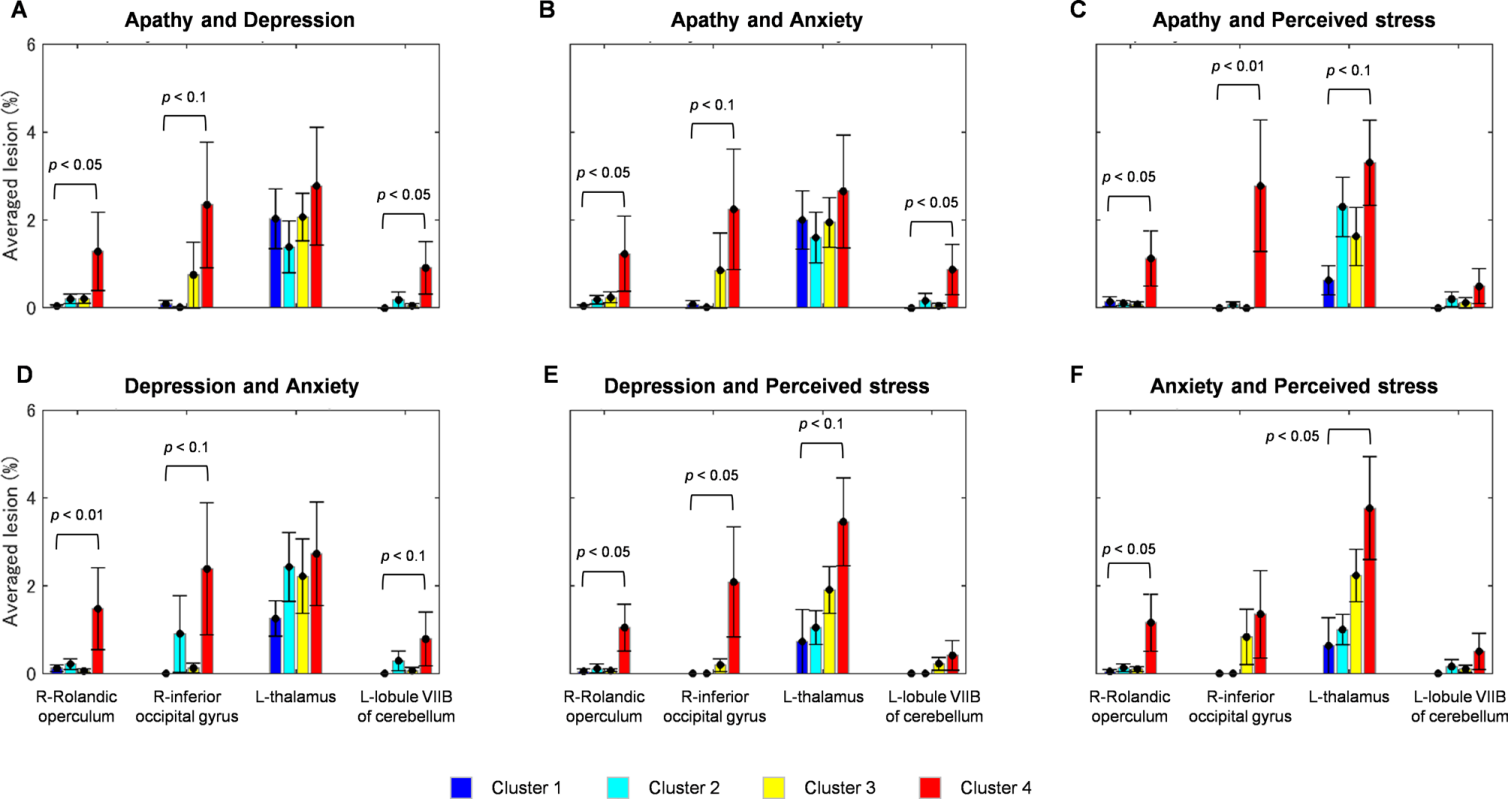
Comparisons of lesion degree at the right Rolandic operculum, right inferior occipital gyrus, left thalamus, and lobule VIIB of left cerebellum between clusters 1 (blue, cyan, yellow, and red bar plots for clusters 1, 2, 3, and 4, respectively) based on apathy–depression (**A**), apathy–anxiety (**B**), apathy–perceived stress (**C**), depression–anxiety (**D**), depression–perceived stress (**E**), and anxiety–perceived stress (**F**) clustering. Error bars represent the standard errors of lesion degree for each cluster.

Figure 4 shows bar plots to evaluating the lesion degree at particular regions (right Rolandic operculum, right inferior occipital gyrus, left thalamus, lobule VIIB of left cerebellum) between clusters. The Tukey–Kramer post hoc analysis was performed on the lesion data of above-mentioned regions, significant differences between cluster 4 and other clusters (i.e., cluster 4 vs. 1, cluster 4 vs. 2, or cluster 4 vs. 3) have also been confirmed (*p* < 0.1). Stroke patients with worse psychological condition (high psychological scores; cluster 4) were associated with greater averaged lesions than those in other clusters (Supplementary Table S1).

## Discussion

In the current study we investigated the relationships between patients’ psychological conditions and brain lesion degrees. The psychological conditions were categorized through a data-driven and unsupervised method, the *k*-means clustering method, without the use of clinical cut-offs. Four clusters were found to appropriately define the psychological conditions of stroke patients. These clusters were then assumed to represent a spectrum of symptoms and severity levels. Lesions in the right Rolandic operculum contributed to worse psychological conditions (high apathy, depression, anxiety, and perceived stress).

Acute stress with sudden rise in blood pressure and/or cerebral blood flow has been reported to cause thalamic hemorrhages^9,10^, and chronic stress exposure could heighten the severity of post-stroke secondary neurodegeneration in the thalamus^11^. Posttraumatic stress disorder (PTSD) patients showed less activation in the thalamus, anterior cingulate gyrus, and medial frontal gyrus than controls did^12^, and their ratio of cortical to subcortical perfusion changed significantly during flashbacks^13^. Stress may be affected as in the theory of brain abnormalities suggested by Duggal^14^. That is, a thalamic infarct disturbing the thalamic amygdala pathway involved in processing fear and traumatic memories might induce the onset of PTSD. We obtained a result that the high lesion degree in the left thalamus was associated with high perceived stress. This result is consistent with previous studies in which stress and thalamus abnormalities (e.g., lesion, hypoactivation, hypo- or hyper-perfusion, etc.) were related to each another.

Meanwhile, the high severity level for other psychological domains (i.e., apathy, depression, and anxiety) was associated with the high lesion degree at the right Rolandic operculum (i.e., post-central operculum). Several studies have reported relationships between the Rolandic operculum and psychology (or emotion) with or without an external factor (disease) and/or intervention (substance abuse). The role of the operculum is also related to emotion processing through music^15,16^ and facial expression^17^. Mean diffusivity of the Rolandic operculum is positively correlated with indices of empathizing-cooperativeness^18^ and depressive symptoms^19^, and hypoactivation in the right parietal and central operculum is associated with impaired emotion processing due to alcohol dependence^20^. Furthermore, hypoactivation was observed not only in the Rolandic operculum but also in the Heschl gyrus, insula, parahippocampal gyrus, posterior cingulate cortex, and inferior frontal gyrus of major depressive disorder (MDD) patients^19^.

Even though the number of study was scarce, it was reported that PSD was associated with cerebellar lesions, including the lobule VIIB of left cerebellum^21^. Effects on the specific left laterality was found and addressed by the asymmetric cerebellar functions^22^. Furthermore, the prefronto-cerebellar network contributes to the function of emotional processing^23,24^; the network disruption may cause the impaired function^25^. A cerebellar damage showed a psychosis-related effect^26^. The pathological mechanism was hypothetically explained by errors of information interpretation and modulation^27^. The errors may extend to the sensory processing, and the damage of sensory-related regions, such as the occipital cortex (vision) also suggested the psychotic condition^28^. The interrelationship between temporoocipital-parietal (sensory) and fronto-limbic-insula (including thalamus) regions proposed the fear network model^29^. This network may explain a manifestation of anxiety illness.

Infarctions in the frontal lobe were observed for patients with post-stroke anxiety (PSA); the occipital infarctions also showed a tendency of higher PSA risk (*p* < 0.1)^30^. The current dataset presented the limited frontal lesions; we were unable to confirm the effect of frontal lesions on psychological domains. Therefore, the effects of worse psychological domains should be evaluated via the brain network concept which its disruptions can be caused by damages (i.e., lesion) of one or more associated regions. The unperformed network analysis may also explain the region inconsistency^31^ found between studies, and those regions might be organized in a network.

Disoriented networks have been associated with psychological symptoms previously. An abnormal increase of connectivity between subgenual prefrontal cortex and the default mode network (DMN) was found in MDD patients^32^. Based on the meta-analysis (five sites with 1,300 patients and 1,128 normal controls), the DMN within-connectivity was found to be reduced for the MDD patients^33^. Therefore, the DMN has also been studied for stroke patients with or without depressive symptoms. Stroke patients reportedly have reduced DMN within-connectivity involving the left medial temporal lobe, posterior cingulate, and medial prefrontal cortices that may be related to the cognitive impairments^34^. Network dysfunctions such as reduce d DMN within-connectivity involving left middle temporal cortex and precuneus^35^ and affective network^36^ have been used to predict the severity of PSD symptoms. Egorova et al.^37^ reported that the network dysfunction related to depressive symptoms was not necessarily associated with network lesions. This result raised a hypothesis of brain plasticity in network recovery. Thus, a future study should address the point of view of connectivity analysis.

Besides playing a role in emotion processing, the Rolandic operculum functions as the sensory system for gustatory and visceral sensation together with the cingulate-operculum network^38,39^. The gastrointestinal tract has been reported being sensitive to emotion (depression, anxiety, stress); the connection between brain and gastrointestinal organs is explained by the theory of a gut-brain axis (top-down and bottom-up)^40^. Negative emotion triggers the excretion of cortisol that induces local immune activity, alters intestinal permeability, and reduces the probiotics level^40,41^. This phenomenon brings two questions—(1) Is controlling parts of the brain (e.g., Rolandic operculum and/or thalamus) feasible for the purpose of modifying negative psychological conditions and specifically easing gastrointestinal symptoms (top-down)? and (2) Is the dietary control more sensitive in suppressing negative psychological conditions (bottom-up)? Answering these questions expectedly brings a strategic treatment for PSD.

The relationship between psychological conditions and lesions has been indicated in the previous and current studies, but the nature of the relationship is still unclear. There are two hypotheses: direct and indirect relationships, respectively drawn from previous results and the current results. The direct relationship suggests that the particular lesion locations may induce worse psychological conditions^14^. Meanwhile, the indirect relationship suggests that the associated disabilities caused by the particular (and other) lesions bring psychological consequences. Patients deny their disabilities, and this denial strongly affects their mental conditions and rehabilitation process^42^. This issue becomes a constraint and our future objective. In order to confirm these hypotheses, a further analysis should be done by investigating the impaired and recovered functions related to the currently found lesions.

Besides, there are two limitations. *First*, the psychological measurement was not set to be the exactly same timing for all patients because the admission timing to the rehabilitation ward depended on the patients’ conditions. Even though the reliability of multiple psychological measurements (e.g. onset and follow-up measurements) was apparently confirmed in the group analysis^43,44^, the relationships between lesions and psychological domains may be dynamic and temporal-dependent within a short interval (1 week vs. 1 month after stroke onset)^43^. Furthermore, personal traits (e.g., neuroticism)^45^ and dependence in activities of daily living (ADL)^46^ highly influenced the development of depressive symptoms over the rehabilitation course. In order to minimize the temporal effects, we excluded patient data with delayed admissions to the rehabilitation ward. However, we did not control the personality factor. A better experimental design should be carried out in a future study. *Second*, the lesion locations were treated here as if they were tightly related to the psychological domains; the laterality effect has never been discussed and should also be addressed further.

In spite of the above mentioned limitations, the current study demonstrated that the risk of worse psychological conditions can be monitored using only the information of lesion degree measured at the initial hospital admission. Comparing its findings with those of previous studies of wide brain lesions, one sees that we have successfully localized robust and specific lesions, aiming at a more accurate evaluation. Therefore, the appropriate treatment for mitigating worse psychological conditions (e.g., PSD) can be incorporated with other rehabilitation courses (e.g., for motor functions), and the speedy recovery of stroke patients is anticipated.

The relationships between psychological conditions of stroke patients and brain lesion degrees were evaluated. Patients with high perceived stress were associated with the high-degree lesions in the left thalamus, whereas other psychological domains (apathy, depression, and anxiety) were significantly associated with high-degree lesions in the right Rolandic operculum. The current findings suggest that using lesion degree analysis to evaluate the risk of worse psychological conditions would help in designing personalized rehabilitation plans for stroke patients.

## Methods

### Subjects

**Figure 5:**
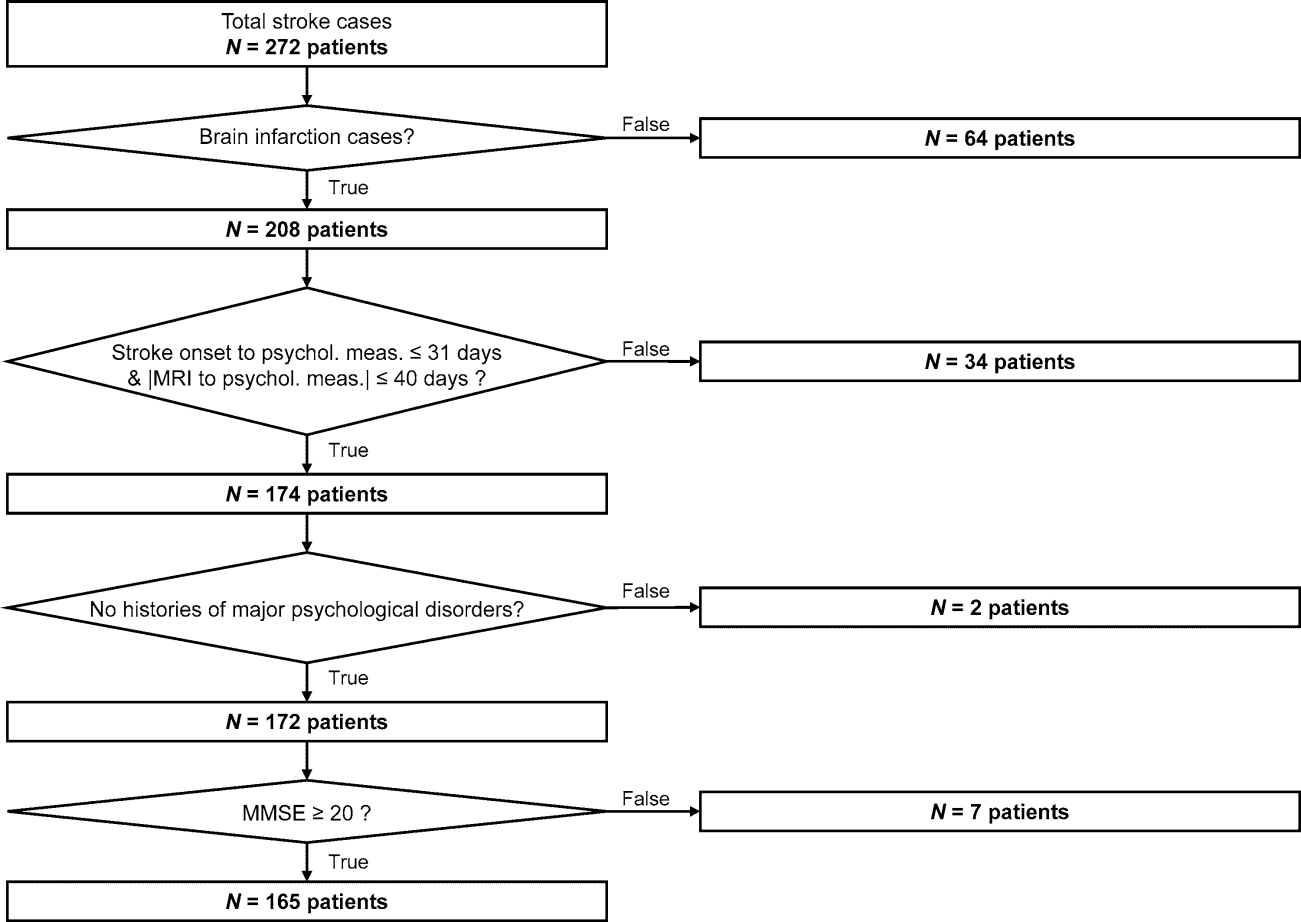
Dataset information with the inclusion criteria.

The current dataset was obtained from a retrospective study. Two hundred seventy-two stroke patients’ data have been collected in the rehabilitation ward of Hibino Hospital, Hiroshima. Two hundred eight patients (170 males; 66.9 ± 9.8 years old; 39–86 years old) were brain infarction cases; the others were either brain (62 patients; 47 males; 60.7 ± 11.5 years old; 34–80 years old) or subarachnoid (2 males; 58- and 65-years old patients) hemorrhage cases. Only brain infarction cases were analyzed in the current study. Patients with severe conditions could not immediately transferred to the rehabilitation ward after the stroke onset. Therefore, the intervals of stroke onset, imaging, and measurement of psychological conditions were widely distributed across patients. In order to minimize the effects of measurement intervals, 34 patients were excluded according to the including criteria: the elapsed time from stroke onset to psychological measurement is equal to or less than 31 days and the time interval between MRI and psychological measurements is equal to or less than 40 days. The only patients without histories of major psychological disorders were analyzed; therefore, two patients with histories of major psychiatric disorders (e.g., major depression disorder, bipolar disorder, schizophrenia, schizoaffective disorder) were also excluded. Even though all patients were able to understand the instructions of performed tests, low Mini-Mental State Assessment scores (less than 20) were found in seven patients. The risk of aphasia might be heightened; thus, those patients were also excluded from the dataset. In total, 165 patient data (134 males; 66.8 ± 9.6 years old; 39–86 years old) were used in the current study. Among 165 patient data, 31 patients had experienced recurrent stroke. Figure 5 shows the dataset information with the inclusion criteria.

Patients’ severities were evaluated from the factors of paresis and limitations in ADL. The six-stage Brunnstrom approach was used to quantify motor functions of lower limbs (five patient data were unavailable; stage 1–2: 6 patients; stage 3–4: 13 patients; stage 5–6: 141 patients), upper limbs (stage 1–2: 6 patients; stage 3–4: 13 patients; stage 5–6: 141 patients), and fingers (stage 1–2: 2 patients; stage 3–4: 12 patients; stage 5–6: 146 patients). Furthermore, the dependency from disabilities was assessed using the Functional Independence Measure (FIM), which was consisted of 18 items (13 and 5 for motor and cognitive items, respectively)^5^. Patients’ disabilities were evaluated by the seven-point scale (scores 1 and 7 for complete dependence and independence, respectively) for each item (126 total FIM score). The total FIM score of the current dataset was 99.3 ± 21.0 (range 36–126). From these factors, patients were mostly in mild-to-moderate severities.

This study was approved by the ethics committee of the Shinaikai Hibino Hospital and the Hiroshima University Epidemiological Research. All experiments were conducted in compliance with relevant regulations and the latest version of the Declaration of Helsinki. Informed consent was obtained from either patients or those authorized to provide consents on behalf of patients, including family members or guardians, before we started any measurements.

### Imaging and non-imaging data

**Figure 6:**
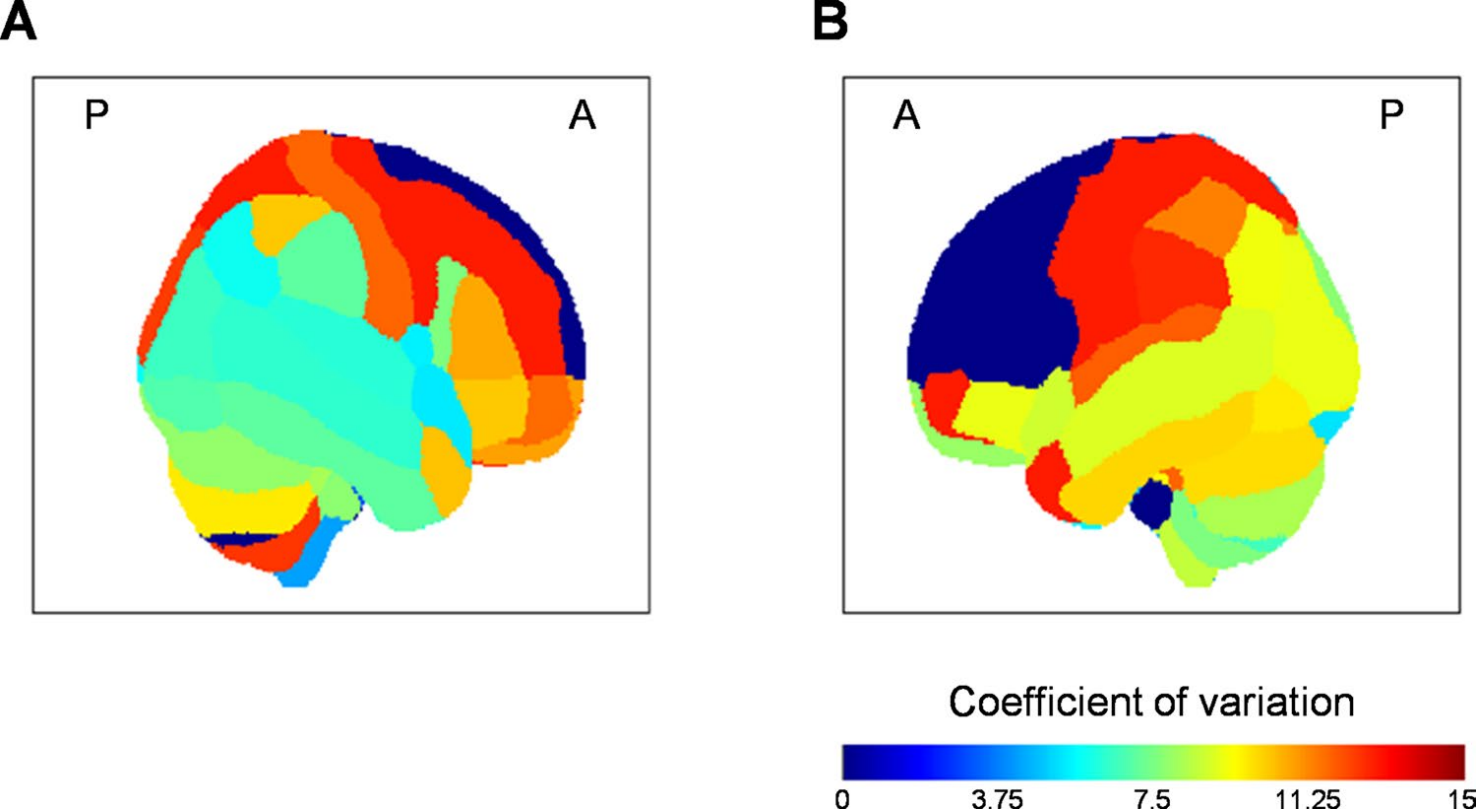
Coefficient of variation map for the lesion data across brain regions depicted from (**A**) right and (**B**) views. A and P represent anterior and posterior, respectively.

Patient data were classified into two types: imaging and non-imaging data. Imaging data were obtained from T2-weighted brain scans using a 1.5-T MRI scanner (Signa EXCITE, XI, ver. 11.0, GE Healthcare, Milwaukee, WI). In order to quantify the brain lesions, five steps were performed. *First*, brain scans were normalized (e.g., non-linear spatial normalization)^47,48^ and standardized across patients following the spatial registration of Montreal Neurological Institute brain template (2 × 2 × 2 mm^3^ voxel size). *Second*, brain lesions were identified by a doctor (KS), and the lesions were manually labeled using MRIcron software (https://www.cabiatl.com/mricron/). The lesion data were not smoothed. The lesion analysis is described in detail elsewhere^4,49,50^. *Third*, brain regions were labeled following the Automated Anatomical Labeling^51–53^ (AAL, i.e., into 116 regions). *Fourth*, lesion and total voxels were calculated for each labeled region. *Fifth*, the ratio between lesion and total voxels (in percentage unit) was computed for each region (This ratio was also referred to here as the “lesion degree measure”). Figure 6 shows coefficients of variation (CV; *σ*/*µ*) for region’s lesions across patients. The low CV value represents low variability across patient, and vice versa. No lesion was observed in some frontal regions (CV = 0). Cognitive data, motor-function data, and psychological data were collected, but in the current study we focused on only the psychological data. Psychological conditions including apathy, depression, anxiety, and perceived stress were assessed using Japanese versions of the Apathy Scale (0–42 range; 12.1 ± 7.3)^3–5,54–56^, Hospital Anxiety (0–21 range; 4.8 ± 3.5) and Depression Scale (0–21 range; 5.1 ± 3.4)^2,4,57,58^, and Perceived Stress Scale (0–56 range; 19.4 ± 7.3)^59,60^. Both imaging and non-imaging data were collected within 1 month after admission to the rehabilitation ward.

### Clustering

Without defining any cut-off for categorizing patients based on psychological conditions, data-driven and unsupervised *k*-means clustering was performed. The input of the *k*-means algorithm could be psychological scores from two domains or more. However, in order to clearly interpret the resulting clusters, patients were evaluated based on combinations of only two psychological domains: apathy–depression, apathy–anxiety, apathy–perceived stress, depression–anxiety, depression–perceived stress, and anxiety–perceived stress. The number of clusters (cluster number) was optimized within a range of 2–10 clusters. The centroid was obtained at each clustering, and good clustering was revealed by a short distance between the centroid and its cluster member (i.e., low intra-cluster similarity) and long distance between the centroid and other cluster members (i.e., high inter-cluster similarity) in the Euclidean space. The explained variance parameter was quantified by the ratio between total variance minus within-cluster variance and total variance. Furthermore, the elbow method^7,8^ was empirically applied on the plot of explained variance against cluster number. Psychological scores of patients were then clustered by the optimum cluster number. Afterwards, each patient would be labelled according to the clusters. The brain lesion degrees were analyzed based on the same clusters. Furthermore, the cluster effect on the brain lesion degree was statistically evaluated using either a two-sample *t*-test or one-way analysis of variance (ANOVA), depending on the cluster number (two, or more than two clusters).

## Supplementary information

Supplementary Table S1.
