# Supplementary material for: Lesions in the right Rolandic operculum are associated with self-rating affective and apathetic depressive symptoms for post-stroke patients
Source: Sci Rep. 2020 Nov 20;10:20264. doi: 10.1038/s41598-020-77136-5 (PMC7679372; doi:10.1038/s41598-020-77136-5)
Supplement: Supplementary file 1 — Supplementary Table S1. [file 41598_2020_77136_MOESM1_ESM.pdf]

Supplementary Information

Title : Lesions in the right Rolandic operculum are associated with self-rating affective and apathetic depressive symptoms for post-stroke patients

Authors : Stephanie Sutoko, Hirokazu Atsumori, Akiko Obata, Tsukasa Funane, Akihiko Kandori, Koji Shimonaga, Seiji Hama, Shigeto Yamawaki, and Toshio Tsuji

Supplementary Table S1. Results of Tukey-Kramer *post hoc* analysis (*p*-values). Bold-written values indicate $p < 0.1$

Domains	Cluster comparisons					
	**1 vs. 2**	**1 vs. 3**	**1 vs. 4**	**2 vs. 3**	**2 vs. 4**	**3 vs. 4**
**Right Rolandic operculum**						
Apathy and depression	0.966	0.959	**0.020**	1.000	**0.045**	**0.050**
Apathy and anxiety	0.972	0.940	**0.025**	0.998	**0.050**	**0.087**
Apathy and perceived stress	1.000	0.998	**0.055**	1.000	**0.025**	**0.042**
Depression and anxiety	0.991	0.999	**0.004**	0.982	**0.019**	**0.009**
Depression and perceived stress	0.999	1.000	0.199	0.999	**0.047**	**0.015**
Anxiety and perceived stress	0.997	0.999	0.116	1.000	**0.025**	**0.017**
**Right inferior occipital gyrus**						
Apathy and depression	1.000	0.839	0.109	0.766	**0.077**	0.357
Apathy and anxiety	1.000	0.783	0.125	0.696	**0.085**	0.487
Apathy and perceived stress	1.000	1.000	**0.009**	1.000	**0.006**	**0.011**
Depression and anxiety	0.586	0.999	**0.058**	0.778	0.496	0.135
Depression and perceived stress	1.000	0.998	0.292	0.993	**0.064**	**0.064**
Anxiety and perceived stress	1.000	0.865	0.663	0.637	0.392	0.930
**Left thalamus**						
Apathy and depression	0.901	1.000	0.922	0.871	0.618	0.927
Apathy and anxiety	0.971	1.000	0.943	0.981	0.779	0.927
Apathy and perceived stress	0.253	0.754	**0.050**	0.891	0.728	0.397
Depression and anxiety	0.455	0.802	0.547	0.970	0.999	0.955
Depression and perceived stress	0.995	0.798	0.195	0.747	0.067	0.298
Anxiety and perceived stress	0.991	0.563	0.092	0.419	0.024	0.383

Supplementary Table S1. continued

Domains	Cluster comparisons					
	**1 vs. 2**	**1 vs. 3**	**1 vs. 4**	**2 vs. 3**	**2 vs. 4**	**3 vs. 4**
**Lobule VIIB of left cerebellum**						
Apathy and depression	0.903	0.998	**0.028**	0.953	**0.097**	**0.034**
Apathy and anxiety	0.916	0.997	**0.035**	0.972	0.103	**0.054**
Apathy and perceived stress	0.869	0.977	0.359	0.990	0.727	0.620
Depression and anxiety	0.608	0.994	**0.056**	0.845	0.471	0.157
Depression and perceived stress	1.000	0.931	0.724	0.797	0.459	0.881
Anxiety and perceived stress	0.971	0.990	0.563	0.997	0.607	0.485
